# Towards High Molecular Weight Furan-Based Polyesters: Solid State Polymerization Study of Bio-Based Poly(Propylene Furanoate) and Poly(Butylene Furanoate)

**DOI:** 10.3390/ma13214880

**Published:** 2020-10-30

**Authors:** Lazaros Papadopoulos, Eleftheria Xanthopoulou, George N. Nikolaidis, Alexandra Zamboulis, Dimitris S. Achilias, George Z. Papageorgiou, Dimitrios N. Bikiaris

**Affiliations:** 1 Laboratory of Polymer Chemistry and Technology, Department of Chemistry, Aristotle University of Thessaloniki, 54124 Thessaloniki, Greece; lazaros.geo.papadopoulos@gmail.com (L.P.); azamboulis@gmail.com (A.Z.); axilias@chem.auth.gr (D.S.A.); 2 Department of Chemistry, University of Ioannina, P.O. Box 1186, 45110 Ioannina, Greece; elefthxanthopoulou@gmail.com (E.X.); geoniknikolaidis@gmail.com (G.N.N.); gzpap@uoi.gr (G.Z.P.)

**Keywords:** biobased polymers, furan dicarboxylic acid, polyesters, poly(propylene furanoate), poly(butylene furanoate), solid state polymerization, thermal properties

## Abstract

In the era of polymers from renewable resources, polyesters derived from 2,5 furan dicarboxylic acid (FDCA) have received increasing attention due to their outstanding features. To commercialize them, it is necessary to synthesize high molecular weight polymers through efficient and simple methods. In this study, two furan-based polyesters, namely poly (propylene furanoate) (PPF) and poly(butylene furanoate) (PBF), were synthesized with the conventional two-step melt polycondensation, followed by solid-state polycondensation (SSP) conducted at different temperatures and reaction times. Molecular weight, structure and thermal properties were measured for all resultant polyesters. As expected, increasing SSP time and temperature results in polymers with increased intrinsic viscosity (IV), increased molecular weight and reduced carboxyl end-group content. Finally, those results were used to generate a simple mathematical model that prognosticates the time evolution of the materials’ IV and end groups concentration during SSP.

## 1. Introduction

In recent years, there is an ever-growing concern to develop bio-based and biodegradable plastics. While traditional petroleum-based polymers have dominated the market for several decades, environmental and sustainability issues have emerged and a shift towards eco-friendly materials has been observed [1]. The main reasons behind this are the fluctuation of crude oil prices, as the production of polymers consumes nearly 10% of the annually produced oil [2] and that uncontrolled disposal has led to accumulation of microplastics in the environment on the so-called “garbage patches” [3]. To resolve these issues, research has focused on the exploitation of biomass, in the form of renewable feedstocks like lignin and carbohydrates, to develop novel polymeric materials [4] through the biorefinery system. Several green monomers have been screened through this process, and among them 2,5 furandicarboxylic acid (FDCA) holds a prominent position.

FDCA is an aromatic bio-based building block produced from catalytic oxidation of 5-hydroxymethylfurfural (HMF) and has been selected as one of the twelve most important renewable monomers [5]. Its structure makes it a potential replacement for terephthalic acid (TPA) and for commercial polymers produced by it, like poly(ethylene terephthalate) (PET), poly(butylene terephthalate) (PBT), and poly(butylene adipate-co-terephthalate) (PBAT) [6]. Furan-based polyesters have shown great potential as packaging materials, due to their superior gas barrier properties and milder processing conditions [7,8] compared to their terephthalic counterparts. Also, their properties can be tuned on-demand via copolymerization as a recent review has clearly demonstrated [9]. However, in order to produce polyesters suitable for films, fibers, or bottles, it is mandatory to obtain high molecular weight materials. A very successful method that has been applied in industrial polymers to increase the molecular weight is solid state polymerization (SSP) [10], which found success with polyesters like PET [11,12,13,14,15] and PLA [16,17,18]. Nevertheless, there is a limited number of studies concerning the application of SSP on furan-based polymers. 

The first study where SSP was applied on an FDCA-based polyester was made by de Jong et al. [7] and the polymer of interest was poly(ethylene furanoate) (PEF). However, the progress was monitored by acid value determination and no actual molecular weight increase was provided for comparison. A later study by Knoop et al. [19] showed that after 72 h of SSP, the molecular weight of PEF reached 83,000 g/mol, a ten-fold increase. Hong et al. [20] presented more moderate results, with the initial intrinsic viscosity of PEF being 0.6 dL/g and reaching 0.72 dL/g after 48 h of SSP. Our group has also published a number of works on SSP of PEF, investigating the effect of different nanoinclusions [21] and different catalysts [22,23,24] on the molecular weight of the polyester. The effect of different catalysts was also studied by Celli et al. [25] and more recently, Gabirondo et al. [26] employed SSP to increase the molecular weight of recycled PEF following the principles of a circular economy. From these works, it became apparent that SSP is of vital importance in order to industrialize and commercialize PEF, as it is a process that allows for a molecular weight increase under mild conditions, thus widening the window of potential applications for one of the most important biobased polyesters.

As encouraging as the results of the above-mentioned studies are, the polymer of interest in all of them was always PEF. While it is one of the most promising, it is not the only polyester of the furan family that shows potential. Poly(propylene furanoate) (PPF) and poly(butylene furanoate) (PBF) have also been the topic of many interesting works, as they show potential for usage in multilayer packaging materials, due to their exceptional gas barrier properties [27]. Although many aspects concerning their synthesis, copolymers, and nanocomposites have been examined [28,29,30,31,32,33,34,35,36], to the best of our knowledge, there is no study at the moment involving SSP and those two biobased polymers. So, in this work PPF and PBF polyesters were synthesized and the feasibility of SSP was examined. The effect of temperature and time on the increase of molecular weight was monitored with experimental values along with theoretical modeling, while complementary data were provided by the means of XRD and DSC.

## 2. Materials and Methods 

### 2.1. Materials 

Dimethylfuran-2,5-dicarboxylate (DMFD) was supplied by Global Chemie (Bangladesh). 1,3-propanediol (99%) and 1,4-butanediol (99%), phenol (99+%), and 1,1,2,2-tetrachloroethane (98+%), used as a mixture for intrinsic viscosity measurements, were purchased from Alfa Aesar, Kandel, Germany. Tetrabutyl titanate(IV) (TBT) catalyst of analytical grade was purchased from Aldrich Co (St. Louis, MO, USA). All other solvents and materials used were of analytical grade. 

### 2.2. Polyesters’ Synthesis 

PPF and PBF polyesters were prepared through a two-step melt polycondensation procedure in a glass batch reactor, as described in previous works of our group [37,38]. According to this procedure, 30.0 g of DMFD (0.16 mol) and 27.3 g of 1,3-propanediol or 32.3 g of 1,4-butanediol (0.36 mmol, 2.2 equivalents) were charged into the reaction flask of the polymerization apparatus and TBT (400 ppm) was added as a catalyst. The apparatus with the reagents was evacuated and filled with N_2_ thrice in order to obtain an inert atmosphere. Under nitrogen flow, the reacting mixture was successively heated at 160 °C, 170 °C, 180 °C, and 190 °C for 1 h at each temperature. During the esterification, methanol distilled out of the reaction. When almost all the theoretical amount of methanol was collected, vacuum was implemented progressively to 5.0 Pa and the temperature was gradually increased to 220 °C, over a period of 20° min in order to remove unreacted diol and to avoid excessive foaming and oligomer sublimation. Stirring speed was increased at 750 rpm and after 2 h at 220 °C, the temperature was further increased to 230 °C for 2 more hours. The obtained polyesters were washed with methanol. 

### 2.3. Solid-state Polycondensation

The procedure of solid-state polymerization took place in an apparatus containing four test tubes, connected to a vacuum line and submersed in a thermostated salts bath, according to Kasmi et al. [23]. 3 g of milled polymer were placed in a glass tube under vacuum (3–4 Pa). The reaction was carried out at a constant temperature of 155, 160 or 165 °C for both materials. The tubes were removed from the bath after 1, 2, 3 and 4 h respectively and the polymers were characterized by means of intrinsic viscosity (IV), carboxyl end-group content, and thermal behavior.

### 2.4. Polyesters’ Characterization 

#### 2.4.1. Intrinsic Viscosity (IV) Measurements 

The intrinsic viscosity of the obtained materials was measured with an Ubbelohde viscometer (Schott Gerate GMBH, Hofheim, Germany) at 25 °C. The samples were dissolved in a 60/40 *w/w* solution of phenol and tetrachloroethane, by heating at 80 °C for 10 min. After cooling, all the samples were filtered through a disposable Teflon filter to exclude possible solid residues. The IV of the polyesters was calculated according to the Solomon–Ciuta Equation (1) of a single point measurement.

(1) $$\left[\eta \right]\;=\;\frac{{\left[2\{\frac{t}{t_{0}}-\ln\left(\frac{t}{t_{0}}\right)-1\}\right]}^{\frac{1}{2}}}{c}$$

where *c* is the solution concentration, *t* the flow time of the solution, and *t*_0_ the flow time of the solvent. The reported values are the average of three measurements. 

#### 2.4.2. Molecular Weight 

IV values [η] were used to calculate the number average molecular weight (Mn) of the PPF and PBF polyester samples, using the Berkowitz Equation (2), as modified by our research group in an earlier work [23]:

Mn = 3.29 × 10^4^[η]^1.54^ (2)

#### 2.4.3. End-group Analysis 

The determination of the carboxyl-end group content of the obtained polyesters was made via titration procedure in a solution of benzyl alcohol/chloroform mixture, according to Pohl’s method [39]. The titration of the solution, using NaOH in benzyl alcohol as a standard solution and phenol red as an indicator, was performed thrice and the mean value of [COOH] was calculated. 

#### 2.4.4. Nuclear Magnetic Resonance Spectroscopy (NMR)

^1^H-NMR spectra of polyesters were obtained on an Agilent 500 spectrometer (Agilent Technologies, Santa Clara, CA, USA) at room temperature. Polymers were dissolved in deuterated trifluoroacetic acid (TFA-d) (5% *w/v* solutions). A total of 16 scans were recorded, and the sweep width was 6 kHz. 

#### 2.4.5. Wide Angle X-ray Diffraction Patterns (WAXD)

X-ray powder diffraction (XRD) patterns were recorded using an XRD-diffractometer (Rigaku-MiniFlex II, Chalgrove, Oxford, UK), with CuKα radiation for crystalline phase identification (λ = 0.15405 Å). All materials were examined over the 2θ range of 5° to 50° through a scan speed of 1°/min.

#### 2.4.6. Differential Scanning Calorimetry (DSC) 

To study the effect of SSP on the materials’ thermal behavior, a Perkin–Elmer Pyris 6 differential scanning calorimeter (DSC) (Waltham, MA, USA) calibrated with Indium and Zinc standards, was used. 7 ± 0.1 mg of each sample were placed in a sealed aluminum pan and heated from 30 to 200 °C (heating rate 20 °C/min, inert atmosphere: N_2_, flow rate 50 mL/min).

## 3. Modeling of the Solid-State Polymerization of PPF and PBF Polyesters Kinetics

### 3.1. Reaction Mechanism

The principal reaction that takes place during the solid-state polymerization of PPF and PBF polyesters is the polycondensation/transesterification of hydroxyl-ended polymeric chains as depicted in Reaction (R1). Other side reactions include esterification (Reaction (R2)), thermal degradation (Reaction (R3)) and further side reactions of vinyl end-groups formed by thermal degradation (Reaction (R4)) [40]. In these equations, k_1_ and k_2_ denote the rate constants for the forward reactions in Reactions (R1) and (R2), respectively. The corresponding equilibrium constants are K_1_ and K_2_. k_d_ is the rate constant of the thermal degradation (Reaction (R3)) and k_v_ refers to the polycondensation of propenyl and butenyl end-groups (Reaction (R4)). Both these reactions are considered irreversible. It should be pointed out that the kinetic constants of the reversed Reactions (R3) and (R4), k_1_’ and k_2_’, have been expressed as a function of the forward rate constants and the equilibrium constants: k_1_’ = k_1_/K_1_ and k_2_’ = k_2_/K_2_. These equations stem from the general mathematical definition of the equilibrium constant: K = k_forward_/k_reverse_.

Polycondensation/transesterifications

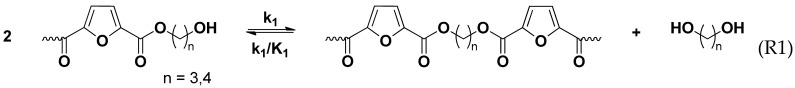



Esterification

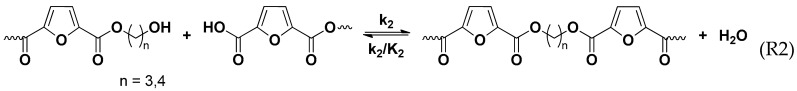



Thermal degradation

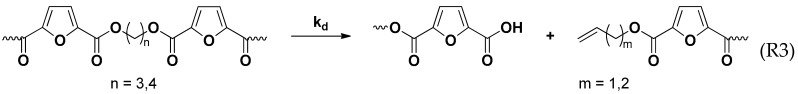



Polycondensation of propenyl and butenyl terminal groups

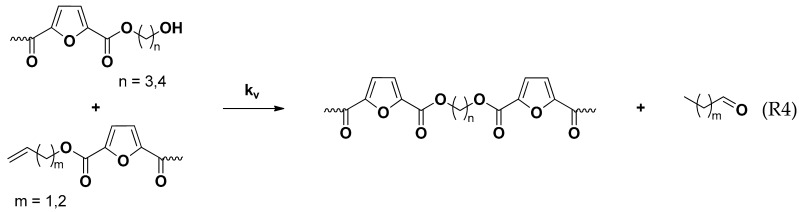



The molecular weight of PPF and PBF polyesters is increased by three reactions. The most important reaction is the polycondensation/transesterification reaction, where two hydroxyl-ended chains react, and propylene or butylene glycol are produced (Reaction (R1)). The second one is the esterification reaction (Reaction (R2)), in which a terminal hydroxyl groups reacts with a carboxyl ended chain and is releasing H_2_O. In contrast, when thermal degradation occurs the molecular weight of the polymer decreases. Generally, polyesters degrade through heterolytic scission, by β-hydrogen abstraction generating a terminal carboxylic acid and a chain ending with a propenyl or butenyl moiety, for PPF and PBF respectively (Reaction (R3)). Finally, the products afforded by thermal degradation can further react with hydroxyl-ended chains, either by esterification (Reaction (R2)) or by transesterification (Reaction (R4)). These reactions result in an increase of the molecular weight. Overall, the reaction rate depends on a combination of intrinsic reaction kinetics, diffusion-related limitations affecting the reactivity of the polymeric chain terminal groups, changes in the degree of crystallization of the polymeric chains, and the diffusion of volatile by-products (i.e., H_2_O and glycols) [15].

### 3.2. Simplified Mathematical Model

Modeling the SSP kinetics is quite complex as, as diffusion limitations must also be taken into account in addition to chemical kinetics that describe how the concentration of the reactive species change during the course of the reaction. As a result, additional variations depending on the distance from the interface must be applied [40]. Thus, a system with two independent variables and a set of partial differential equations, including several diffusional, kinetic, and crystallization parameters, is necessary to describe the reaction [41]. However, there is no physical meaning in utilizing complicated models and a much simpler kinetic modeling approach was adopted, similar to the one developed by Agarwal et al. [42,43]. While this model was initially developed for the SSP of PET, our group has applied it successfully for modeling the SSP of pristine PEF, along with nanocomposite PEF and PET [13,15,21,44].

The mathematical model was established by making the following assumptions:

- The kinetic rate constants are regarded to be independent of the length of the polymer chains (only the reactivity of end-groups is considered).
- As a high vacuum is applied (below 3–4 Pa), the respective glycols and water are removed very quickly from the reaction mixture. As a result, reverse reactions in Reactions (R1) and (R2) are eliminated.
- The relatively low temperatures (i.e., 155–165 °C) where polycondensation occurs, allow us to neglect any thermal degradation reactions (Reaction (R3)), thus excluding Reaction (R4) as well.
- Limitations linked to the diffusion of volatile species are ignored.

Once those simplifications are accepted, the rate of change of the concentrations of hydroxyl ([OH]) and carboxyl ([COOH]) end-groups can be described by Equations (3) and (4) [42,43]:

(3) $$\frac{d{\left[\mathrm{OH}\right]}_{t}}{dt}=-2k_{1}{\left[\mathrm{OH}\right]}_{t}^{2}-k_{2}[\mathrm{COOH}{]}_{t}[\mathrm{OH}{]}_{t}$$

(4) $$\frac{d{\left[\mathrm{COOH}\right]}_{t}}{dt}=-k_{2}[\mathrm{COOH}{]}_{t}[\mathrm{OH}{]}_{t}$$

where [COOH]_t_ and [OH]_t_ denote the actual “real” carboxyl and hydroxyl end-groups concentration, respectively.

Agarwal and co-workers [42,43] introduced “actual hydroxyl and carboxyl end-groups” to account for the rapid drop in SSP kinetics at increased IV values. Accordingly, it was considered that part of the terminal groups was temporarily inactive (these are designated as [OH]_i_ and [COOH]_i_ in Equations (5) and (6). As a result, the active concentration of OH and COOH in Equations (3) and (4) can be expressed as:

(5) $${\left[\mathrm{OH}\right]}_{t}=\left[\mathrm{OH}\right]-{\left[\mathrm{OH}\right]}_{i}$$

(6) $${\left[\mathrm{COOH}\right]}_{t}=\left[\mathrm{COOH}\right]-{\left[\mathrm{COOH}\right]}_{i}$$

where [COOH], [OH] and [COOH]_i_, [OH]_i_ stand for the of the total and temporarily inactivated concentrations of COOH and OH groups respectively. 

Moreover, the end-groups concentration can be used to determine the number average molecular weight:

(7) $$\bar{M_{n}}=\frac{2}{\left[\mathrm{COOH}\right]+\left[\mathrm{OH}\right]}$$

When combined with Equations (2), (5)–(7), Equations (3) and (4) are ordinary differential equations which can be solved using the varying step-size Runge–Kutta method. The solution of those equations will indicate how the IV and the concentration of carboxyl and hydroxyl end-groups change as a function of time during the course of SSP. The values of those three variables were fitted to the experimental data points, resulting to four adjustable parameters, namely [OH]_i_, [COOH]_i_, k_1_ and k_2_, for each experimental condition. 

## 4. Results and Discussion

### 4.1. Synthesis and Characterization of PPF and PBF Polyesters

**Figure 1:**
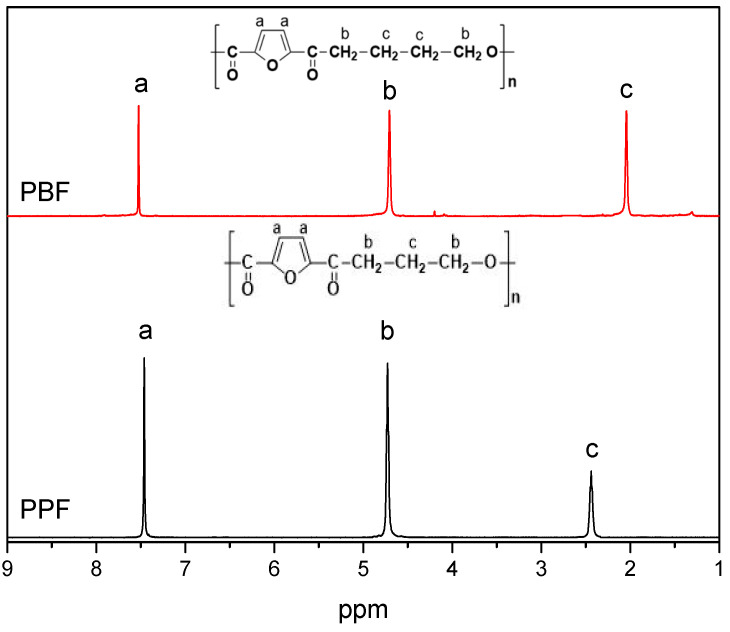
^1^H NMR of the prepared polyesters.

Poly(propylene furanoate) and poly(butylene furanoate) polyesters were synthesized via a two-stage polycondensation reaction with TBT as a catalyst, as aforementioned in the experimental part. The ^1^H NMR spectra shown in Figure 1 prove the successful synthesis as they confirm the structure of the produced materials [37,38]. 

**Figure 2:**
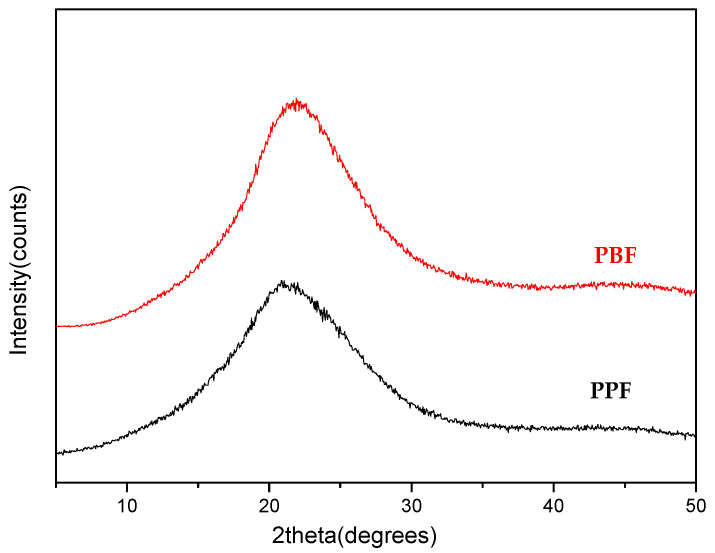
WAXD patterns of the prepared poly(propylene furanoate) and poly(butylene furanoate) polyesters after melt polycondensation procedure.

The obtained materials exhibit intrinsic viscosity values of 0.24 dL/g and 0.53 dL/g. Furthermore, they were completely amorphous as it can be observed in Figure 2. Before SSP procedure, both polyesters were annealed overnight at 100 °C to increase their crystallinity and remove any water that could interfere with SSP. 

### 4.2. Kinetic Study of the Solid-State Polymerization of PPF and PBF Polyesters

SSP is a promising technique widely applied post melt polymerization, aiming at increasing the molecular weight of the polymers and thus improving their mechanical properties and rheological behavior. The ambition of the current work is to rationalize how temperature and time influence the increase of the molecular weight during SSP for PPF and PBF polyesters. In parallel, the effect of reaction temperature and time on the end groups concentration and the intrinsic viscosity of PPF and PBF was also investigated.

**Figure 3:**
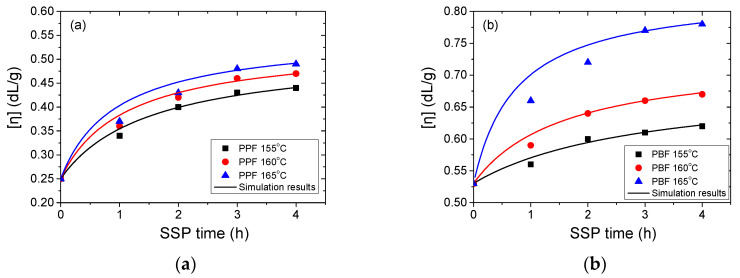
Evolution of the intrinsic viscosity (IV) with time during SSP of (**a**) PPF and (**b**) PBF polyesters at different temperatures. Simulation results are represented by the continuous lines.

SSP for both materials was carried out at 155, 160, and 165 °C for 1, 2, 3, and 4 h. The first variable examined was intrinsic viscosity. Results are given in Figure 3. As it can be observed, regardless of the polyester, the IV increases significantly with increasing time or temperature. For PPF, the IV values start from 0.25 dL/g to attain 0.44 dL/g at 155 °C, 0.47 dL/g at 160 °C and 0.49 dL/g at 165 °C after 4 h of SSP. Similar for PBF, IV started from 0.53 dL/g to reach 0.62, 0.67, and 0.78 dL/g after 4 h of SSP at 155, 160, and 165 °C. The observed trends in IV values give a first indication about the reaction rates for the studied polyesters. As expected, increasing the reaction temperature clearly favors the IV increase, since both transesterification and esterification are accelerated. These reactions are regulated by the diffusion of the respective glycol and water, and as the SSP temperature decreases, the diffusion of the glycol becomes slower. This explains the slow increase of IV at 155 and 160 °C, regardless of polyester type. 

Nevertheless, at 165 °C, a temperature closer to the melting points of PPF and PBF polyesters, a rapid rise of the intrinsic viscosity values was observed during the first two hours. From the increased temperature, the mobility of the macromolecular chains of PPF and PBF is enhanced, resulting in an increased reactivity of the hydroxyl and carboxyl end-groups, and, in turn, higher molecular weights. It can be thus concluded that temperature is one of the most critical parameters during SSP. The Mn of PPF and PBF polyesters samples and the corresponding average degrees of polymerization are presented in the following table (Table 1). These values were calculated from the experimental IV values using Equation (2). Not surprisingly, Mn and IV depend on time and reaction temperature. It should be noted that, since Equation (2) is only available for PEF in the literature, there might be a small approximation in the values calculated for PPF and PBF.

**Figure 4:**
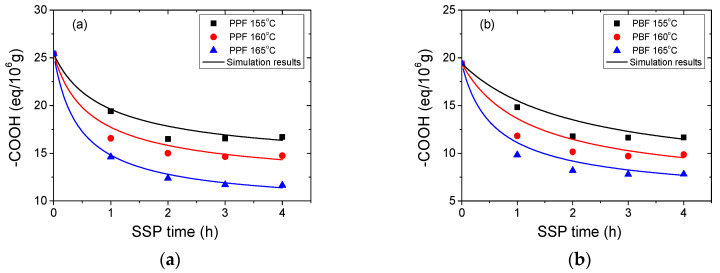
Evolution of carboxyl (–COOH) end-groups concentration with time during SSP of (**a**) PPF and (**b**) PBF polyesters at different temperatures. The theoretical data obtained by the kinetic model simulation are represent by the continuous lines.

The second variable used to monitor the impact of SSP time and temperature is the terminal carboxylic groups (–COOH). Figure 4 displays the effect of reaction temperature and time during SSP on the carboxyl end-groups concentration for PPF and PBF samples. It is important to note that, in the initial PPF and PBF the concentration of carboxyl end-groups is rather small. Indeed, both PPF and PBF polyesters were prepared by the transesterification of DMFD. In the first stage of the polymerization, bis(hydroxypropylene) furanoate and bis(hydroxybutylene) furanoate, which both have only hydroxyl end-groups are formed. The presence of carboxyl end-groups is due to decomposition reactions, similar to Reaction (R3), that takes place during melt polycondensation [45]. Once formed, the carboxyl end-groups can react by esterification (as depicted in Reaction (R2)) contributing to the increase of molecular weight. 

As it can be observed from the experimental data in Figure 4, the concentration of carboxyl end-groups decreases with time and increasing temperature. The trend is more obvious at 165°, which is closest to the melting points of the studied polyesters. For PPF, the initial carboxyl end-groups concentration is 25.43 eq/10^6^ and reaches 11.64 eq/10^6^ after 4 h at 165 °C. For PBF, the carboxyl end-groups concentration begins at 19.41 eq/10^6^ to attain 7.81 eq/10^6^ in similar conditions. The carboxyl group reduction rate is more significant at 165 °C compared to 160 and 155 °C, where the curves were less sharp and almost linear. To conclude, the evolution of the carboxyl end-groups concentration depends essentially on time: there is a continuous downward trend with increasing time, whereas the variation is less significant when increasing the temperature.

**Figure 5:**
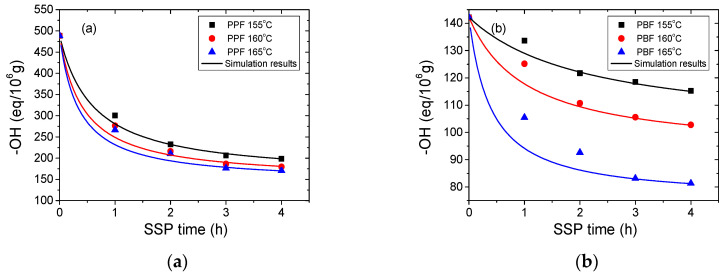
Evolution of hydroxyl (–OH) end-groups concentration with time during SSP of (**a**) PPF and (**b**) PBF polyesters at different temperatures. Simulation results are represented by the continuous lines.

The third variable used to elucidate the influence of time and temperature on SSP is the hydroxyl (–OH) end-groups concentration. Figure 5 presents how the concentration of hydroxyl (–OH) end-groups varies with reaction temperature and time during SSP for PPF and PBF. Predictably, and in accordance with the aforementioned results, the concentration of hydroxyl end-groups decreases with time and the most important decrease was recorded at the highest studied SSP temperature (165 °C). Figure 5 illustrates a rapid reduction in the calculated hydroxyl end-group concentration during the first 2 h for both PPF and PBF polyesters, which becomes slower afterwards. This observation is in good agreement with the conclusions drawn from the IV values, i.e., a significant IV increase during the first 2 h corresponding to an essential increase in the Mn. For PPF the hydroxyl end-groups concentration starts approximately from 488.62 eq/10^6^ to reach 170.72 eq/10^6^ after 4 h at 165 °C, while for PBF the corresponding values are 142.20 eq/10^6^ initially and 81.32 eq/10^6^ finally. 

The resulting simulation curves of the simplified mathematical kinetic model are depicted by the continuous lines in Figure 3, Figure 4 and Figure 5. The observed discrepancies are attributed to the assumptions made when the simplified mathematical model was developed. To conclude, during SSP of PPF and PBF polyesters the mobility of the end-groups was increased, and that lead to the chain extension of the polymers.

Furthermore, the simplified mathematical model presented in the previous section was used to determine the kinetic rate constants of the polycondensation/transesterification and esterification reactions, k_1_ and k_2_, respectively. As described in Section 3.2, differential Equations (3) and (4) were solved affording theoretical values for IV along with end-group concentrations as a function of time during SSP. The parameters of the simplified mathematical model k_1_, k_2_, [OH]_i_ and [COOH]_i_ were calculated by fitting the experimental data. The optimized values of these parameters are summarized in Table 2.

**Figure 6:**
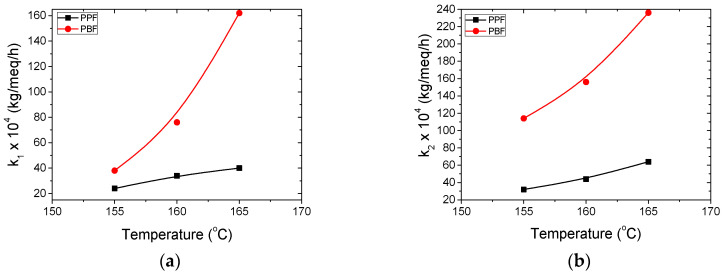
Increase of the estimated kinetic rate constants of (**a**) polycondensation/transesterification (k_1_) and (**b**) esterification (k_2_) reactions with temperature of PPF and PBF polyesters.

According to the data presented in Table 2, the values of the esterification rate constant, k_2_, are higher than that of the transesterification rate constant, k_1_, for both PPF and PBF polyesters. This result is reasonable considering that end groups concentrations are taken into account and measurements showed [–COOH] to be significantly lower than [–OH]. It should be pointed out that PBF exhibited higher polycondensation/transesterification (k_1_) and esterification (k_2_) kinetic rate constants compared to PPF polyester at all reaction temperatures. The above are illustrated in Figure 6.

It is obvious that the temporarily inactivated hydroxyl end-groups, [OH]_i_, are always lower in PBF compared to PPF polyester, while the trend is always a decrease with increased temperature, as the end-groups mobility is enhanced. The lower values of [OH]_i_ in PBF are a direct consequence of the terminal hydroxyl groups concentration which is consistently lower in PBF compared to PPF regardless the temperature or duration of the reaction, as illustrated in Figure 3. Concerning the temporarily inactivated carboxyl end-groups [COOH]_i_, the values are similar for both PPF and PBF polyesters. 

**Figure 7:**
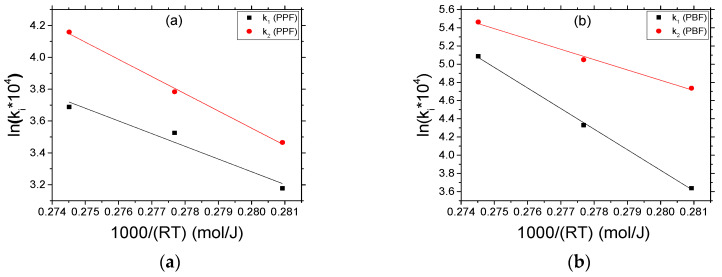
Arrhenius type plots in order to determine the activation energies and the pre-exponential factors of the polycondensation/transesterification (k_1_) and esterification (k_2_) reactions of PPF (**a**) and PBF (**b**) polyesters.

Finally, an Arrhenius-type expression was used to correlate both kinetic rate constants with temperature. Again, enhanced mobility leads to increased kinetic rate constants. Figure 7 illustrates the Arrhenius type plots ln(k) vs. 1000/RT. The activation energies and pre-exponential factors for the polycondensation/transesterification, E_1_, and esterification, E_2_, reactions were graphically determined from the slope and the intercept of the lines in the obtained diagrams. These parameters, along with the correlation coefficients for both PPF and PBF polyesters, are summarized in Table 3.

It should be observed that some uncertainty is included when estimating the activation energies for the transesterification and esterification reactions using so few experimental data points (at each investigated temperature), as denoted by the rather high standard deviation values. Thus, a safe conclusion cannot be drawn. However, there is a definite indication that for the polycondensation/transesterification reaction (Reaction (R1)), PPF has a much lower activation energy E_1_ compared to PBF and the activation energy E_2_ for the esterification reaction (Reaction (R2)) seems to be also lower (within experimental error). 

### 4.3. Thermal Analysis of Polyesters after SSP

**Figure 8:**
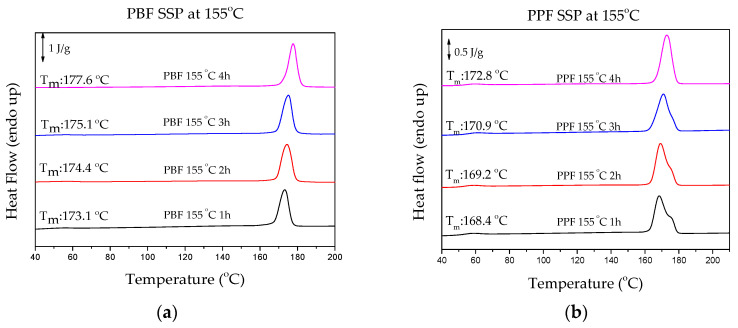

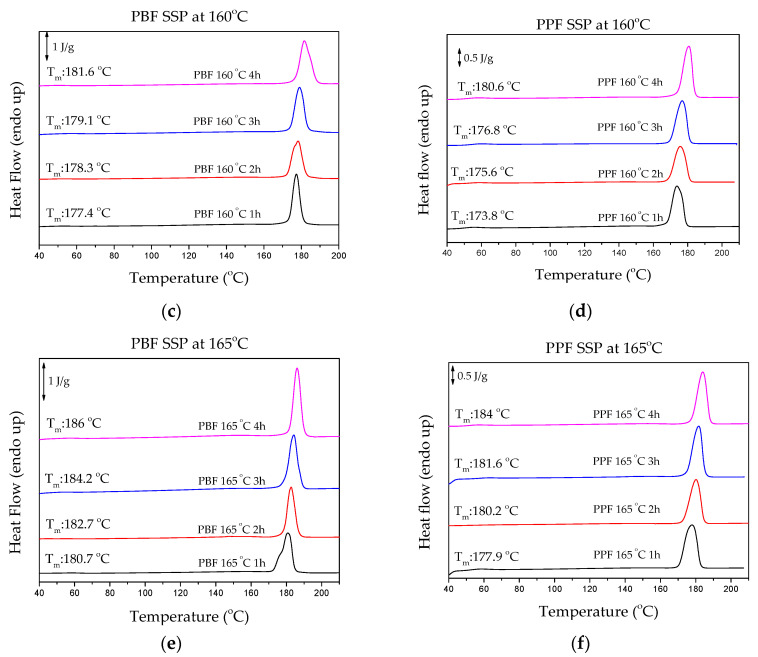
DSC thermograms of the prepared polyesters after SSP at 155 °C for (**a**) PBF, (**b**) PPF, after SSP at 160 °C for (**c**) PBF, (**d**) PPF and after SSP at 165 °C for (**e**) PBF, (**f**) PPF.

The thermal properties of PPF and PBF polyesters were studied with DSC. The results shown in Figure 8 clearly depict that the time and temperature of SSP influence them greatly. Both melting temperature and crystallinity reach higher values with increasing SSP time and temperature, following the increase of the molecular weight as analyzed above. 

**Figure 9:**
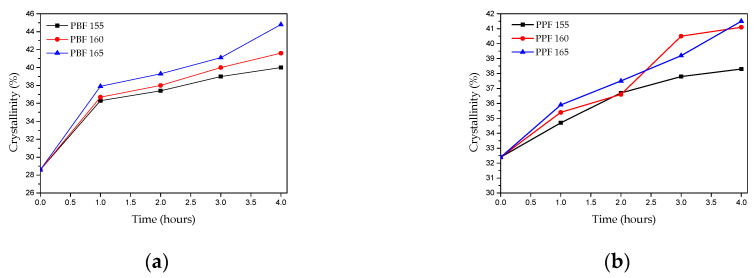
Evolution of the crystallinity degree (X_c_) with time and temperature of SSP for (**a**) PBF and (**b**) PPF.

The degree of crystallinity (Xc) of all samples in this study are shown in Table 4. The experimental melting enthalpy (∆H_m_) calculated from DSC and the heat of fusion of the pure crystalline PPF and PBF [37,38] were used to calculate it. Interestingly, as depicted in Figure 9, PBF reached a higher crystallinity degree compared to PPF (44.8 and 41.5 respectively), though, the starting degree of crystallinity was lower for PBF (28.7 and 32.4 for PBF and PPF, respectively). This finding supports the claim that SSP takes place in the amorphous regions of the polymer. As crystallinity greatly influences the diffusion rate and the mobility of the polymer chain end-groups, which are located in the amorphous phase of semi-crystalline polyesters, it can be deduced that the rapid increase in molecular weight of PBF is linked to its lower degree of crystallinity, as presented in Figure 9. The unwanted byproducts were removed more easily, thus the molecular weight increased faster. This assertion is also in agreement with the Mn/IV values obtained in the present study and the findings of studies concerning SSP of PEF [23].

## 5. Conclusions

This study involves the investigation of the feasibility of SSP procedure to biobased polyesters PPF and PBF. To the best of our knowledge, it is the very first work involving a furan-based polyester other than PEF. As expected, the IV and Mn of both materials increase with SSP time and temperature, as the elimination of the byproducts formed in the course of the reaction is diffusion controlled. The SSP kinetics of PPF and PBF were investigated at 155, 160, and 165 °C, both experimentally and by simple kinetic modeling. A simple kinetic model was applied, in order to describe the evolution of IV, hydroxyl and carboxyl content during the SSP of the studied polyesters. Both experimental measurements and theoretical simulation data demonstrated that PBF possesses a higher transesterification kinetic rate constant, i.e., a higher reaction rate, compared to PPF polyester, a claim further verified by the difference of concentration of temporarily inactivated end-groups between the two materials. 

## Figures and Tables

**Table 1:** Number average molecular weights (Mn, g/mol) of PPF and PBF polyesters obtained after SSP at different temperatures and times. The average degree of polymerization is also included in the parenthesis.

Temperature (°C)	SSP Time (h)	PPF	PBF
as received	0	3890 (20)	12380 (59)
155	1	6660 (34)	13800 (66)
	2	7990 (41)	14700 (70)
	3	8780 (45)	15400 (73)
	4	9310 (48)	15800 (75)
160	1	7500 (38)	15200 (72)
	2	8960 (46)	16500 (79)
	3	9760 (50)	17300 (82)
	4	10300 (53)	17800 (85)
165	1	8110 (41)	19000 (90)
	2	9680 (49)	21000 (100)
	3	10500 (54)	21900 (104)
	4	11000 (56)	22500 (107)

**Table 2:** Kinetic rate constants of the polycondensation/transesterification (k_1_) and esterification (k_2_) reactions and concentration of temporarily inactivated OH and COOH end-groups at different reaction temperatures for PPF and PBF polyesters.

Sample	Temperature (°C)	[OH]_i_ (meq/kg)	[COOH]_i_ (meq/kg)	k_1_·(kg/meq)·h^−1^	k_2_·(kg/meq)·h^−1^
PPF	155	155	13	24 × 10^−4^	32 × 10^−4^
	160	149	11	34 × 10^−4^	44 × 10^−4^
	165	143	9	40 × 10^−4^	64 × 10^−4^
PBF	155	100	7.5	38 × 10^−4^	114 × 10^−4^
	160	93	5.5	76 × 10^−4^	156 × 10^−4^
	165	76	4	162 × 10^−4^	236 × 10^−4^

**Table 3:** Activation energies, pre-exponential factors and correlation coefficients of the polycondensation/transesterification and esterification reactions of PPF and PBF polyesters.

Sample	E_1_ (kJ/mol)	ln(k_1_ × 10^4^)	R^2^	E_2_ (kJ/mol)	ln(k_2_ × 10^4^)	R^2^
PPF	79.78 ± 6.20	25.62 ± 4.50	0.921	108.07 ± 5.78	33.81 ± 1.61	0.994
PBF	226.10 ± 7.24	67.14 ± 2.01	0.998	113.42 ± 9.79	36.58 ± 2.72	0.985

**Table 4:** Crystallinity degree (%) of PPF and PBF samples after SSP.

SSP Temperature (°C)	SSP Time (h)	PPF	PBF
	0	32.4	28.6
155	1	34.7	36.3
	2	36.7	37.4
	3	37.8	39
	4	38.3	40
160	1	35.4	36.7
	2	36.6	38
	3	40.5	40
	4	41.1	41.6
165	1	35.9	37.9
	2	37.5	39.3
	3	39.2	41.1
	4	41.5	44.8
